# Temperature Dependency on the Microscopic Mechanism in the Normal Direction of Wrought AZ31 Sheet under Dynamic Compressive Behavior

**DOI:** 10.3390/ma14237436

**Published:** 2021-12-03

**Authors:** Feng Zhang, Mingcheng Sun, Baojie Sun, Fengzheng Zhang, Yikui Bai, Zheng Liu

**Affiliations:** 1 School of Water Conservancy, Shenyang Agricultural University, Shenyang 110866, China; Fzhang2019@syau.edu.cn (F.Z.); zhangfz1997@126.com (F.Z.); 2 State Grid Liaoning Electric Power Research Institute, Shenyang 110006, China; sunmingcheng007@sina.com; 3 Anshan Solenoid Valve Co., Ltd., Anshan 114300, China; sun-baojie@163.com; 4 School of Material Science and Engineering, Shenyang University of Technology, Shenyang 110870, China; cl0804zf@126.com

**Keywords:** wrought AZ31 magnesium alloy, dynamic compressive behavior, Schmid Factor, deformation mechanism

## Abstract

In order to analyze the competitive relationship of different deformation mechanisms in wrought AZ31 magnesium alloy, the dynamic compressive experiments were conducted by a Split Hopkinson Pressure Bar (SHPB) apparatus and a resistance-heated furnace in the range of temperature between 20 and 350 °C at the strain rate of 1000 s^−1^. With the help of Electron Backscattered Diffraction (EBSD) observation, theoretical calculated Schmid Factor (SF), Critical Resolved Shear Stress (CRSS), and critical equivalent stress (σ0.2), the dynamic compressive deformation behavior and corresponding mechanism of wrought AZ31 magnesium alloy along the normal direction (ND) were revealed in the current study. The results demonstrate that the c-axis of grains are gradually reoriented parallel to the normal direction of wrought AZ31-ND sheet with the temperature increasing, except the dynamic recrystallization (DRX) mechanism was activated or grains grew up. The non-basal slip and $\left\{10\bar{1}2\right\}$ tension twinning are respectively the predominant deformation mechanisms at lower temperatures (≤250 °C) and higher temperatures (≥250 °C). The predominant type of DRX mechanism of wrought AZ31-ND sheet is rotational dynamic recrystallization (RDRX), which is regarded as an obstacle for the kernel misorientation concentration region enhancement.

## 1. Introduction

The development of a wide range of structural and functional materials for energy generation, energy storage, propulsion, and automotive industry is promoted by the compelling useful need for lightweight, energy-efficient, and environmentally begin engineering systems. As a result of weight savings, translating to lower energy consumption, magnesium alloys, with a density of about 35% and 77% less than that of aluminum alloy and steel, respectively, are widely applied in automotive, aircraft, and aerospace to enhance energy efficacy [1]. In the matter of mechanical, chemical, and physical properties, magnesium alloy is the most complex among the widely available metallic alloys that form the basis of structural engineering material, especially wrought AZ31 magnesium alloy with a strong texture [2]. As a result of hexagonal-close-packed (HCP) Mg-deforming plastically in the crystallographic <c> direction, the dislocation glide on the pyramidal II plane with the <c+a> Burgers vector is a major contributor to c-axis strain [3]. Hence, the <c+a> dislocation slip partly coincides with the inability of wrought AZ31 magnesium alloy to achieve high plastic strain. However, owing to the HCP structure of wrought AZ31 magnesium alloy and due to a lack of independent slip systems, twinning is regarded as an important deformation mechanism at room temperature, which consists of $\left\{10\bar{1}2\right\}$ tension twinning and $\left\{10\bar{1}1\right\}$ contraction twinning [4,5], especially in high strain rate deformation. In addition, when the temperature, strain rate, and plastic strain are simultaneously satisfied to a critical value, the dynamic recrystallization (DRX) would be correspondingly activated [6,7,8]. Particularly, when conducting dynamic compression deformation behavior under a simultaneously high temperature and high strain rate, the deformation mechanisms of non-basal slip and twinning are unusual, resulting in a confounding, conflicting, and mechanistically unexplained phenomenon connected to the anisotropy mechanical properties of wrought AZ31 magnesium alloy with strong $\left\{0002\right\}$ texture [9,10,11]. Hence, uncovering the evolution of non-basal slip, twinning, and DRX mechanisms is thus the key issue regarding the weak anisotropy mechanical properties of wrought AZ31 magnesium alloy with a strong $\left\{0002\right\}$ texture during dynamic compression deformation under high temperatures.

Because of its critical importance and promise, the anisotropy mechanical response of magnesium and its alloys, especially wrought AZ31 sheet, has been extensively investigated at different strain rates and temperatures [6,7,12,13]. Due to the different activation critical conditions and mechanical response segment of non-basal slip, $\left\{10\bar{1}2\right\}$ tension twinning, and $\left\{10\bar{1}1\right\}$ contraction twinning, the shapes of stress–strain curves obviously show two types, such as sigmoidal (concave up) and power-law (concave down), as a result of different strain rates, temperatures, and loading directions. Particularly, the loading direction and temperature are factors that strongly determine the shape of mechanical curves, which has a significant effect on the microscopic deformation mechanism [12,14]. As a result of obvious characteristics about the strong $\left\{0002\right\}$ texture in wrought AZ31 magnesium alloy, many studies have focused on the loading direction perpendicular to the c-axis of grains [5,7,14,15,16,17,18,19,20,21,22,23,24]. When the loading direction is almost perpendicular to the normal direction of wrought AZ31 magnesium alloy sheet (AZ31-ND) at room temperature, the average Schmid Factors (SF) of different microscopic deformation mechanisms are 0.439 (pyramidal <a> slip), 0.427 (pyramidal <c+a> slip), 0.422 (prismatic slip), 0.412 ($\left\{10\bar{1}2\right\}$ tension twinning), 0.203 (basal slip), and almost 0 ($\left\{10\bar{1}1\right\}$ contraction twinning) [5,15]. In addition, the critical resolved shear stress (*CRSS*) of pyramidal slip, prismatic slip, $\left\{10\bar{1}2\right\}$ and tension twinning is 39.2, 45–81, and 2.0–2.8 MPa, respectively [14,16]. Hence, the $\left\{10\bar{1}2\right\}$ tension twinning is almost activated early rather than other microscopic deformation mechanisms, which is particularly significant for the strain softening effect on the initially mechanical response of wrought AZ31 magnesium alloy. Moreover, when the temperature is correspondingly increased, the *CRSS* of pyramidal slip, prismatic slip, and $\left\{10\bar{1}2\right\}$ tension twinning is sharply decreased. The *CRSS* of non-basal slip is even equivalent or smaller than the one of $\left\{10\bar{1}2\right\}$ tension twinning [7]. Hence, the priority activation of $\left\{10\bar{1}2\right\}$ tension twinning is gradually weakened, and the stress–strain curve of wrought AZ31 magnesium alloy is converted from sigmoidal (concave up) to a power-law (concave down) [17,18,19]. During the dynamic deformation of wrought AZ31 magnesium alloy at high temperatures, the dynamic recrystallization mechanism is partly activated, which can be classified into two types: twin-induced dynamic recrystallization (TDRX) and continuous dynamic recrystallization (CDRX) [20]. For the TDRX mechanism, the mutual intersection of primary twins, the occurrence of secondary twinning, and the subdivision into nuclei by the development of traversing low-angle grain boundaries inside large twin lamellae into high-angle grain boundaries depending on the strain increase are significantly determined factors for the formation of dynamic recrystallization [21,22]. For the CDRX mechanism, rotational dynamic recrystallization (RDRX) is the most common type in magnesium alloys, which is mainly caused by local lattice rotation due to dislocation accumulation, leading to the transformation of the grain boundary from a low-angle grain boundary to a high one [18]. Otherwise, when the density of twins and the loading condition simultaneously satisfy the certain critical status, the detwinning mechanism is occasionally activated [23,24]. Hence, $\left\{10\bar{1}2\right\}$ tension twinning plays a very important role in the deformation mechanism of wrought AZ31 sheet. Hence, almost all investigations of wrought AZ31 sheet have completely focused on the evolution of mechanism deformation and microstructure observation in the direction of impact loading perpendicular to the c-axis.

In our current study, the dynamic compressive behavior of wrought AZ31-ND sheet was investigated in the temperature range between 20 and 350 °C at the strain rate of 1000 s^−1^. The high strain rate and elevated temperature compressive tests were carried out with the Split Hopkinson Pressure Bar (SHPB) apparatus and a resistance-heated furnace. After discussing the activation and evolution of the microstructure deformation mechanism in wrought AZ31-ND sheet, such as basal slip, pyramidal <a> slip, pyramidal <c+a> slip, prismatic slip, $\left\{10\bar{1}2\right\}$ tension twinning and $\left\{10\bar{1}1\right\}$ contraction twinning by electron backscattered diffraction (EBSD) observation, Schmid Factors (SF) statistics, and Critical Resolved Shear Stress (*CRSS*) statistics, the present study provides the necessary information required to understand the complicatedly competitive relationship among the microstructure deformation mechanisms in wrought AZ31-ND sheet.

## 2. Experimental

### 2.1. Experimental Material

The experimental material was wrought AZ31 sheet, of which the chemical composition is presented in Table 1. The wrought AZ31 sheet (with an average grain size of ~25 μm) was produced by multi pass under 450 °C with a thickness of 8 mm, which was produced by Timminco Metals in Denver, CO, USA. As large twins were generated during the AZ31 rolled sheet production, the detwinning process was necessary to avoid negative effects on the microstructure deformation mechanism analysis. The heat treatment process of detwinning was 300 °C × 2 h. The size of the test sample was Φ6 × 6 mm, which was cut along the normal direction of the AZ31 rolled sheet by using the electrical discharged wire-cutter.

### 2.2. Experimental Method

**Figure 1:**
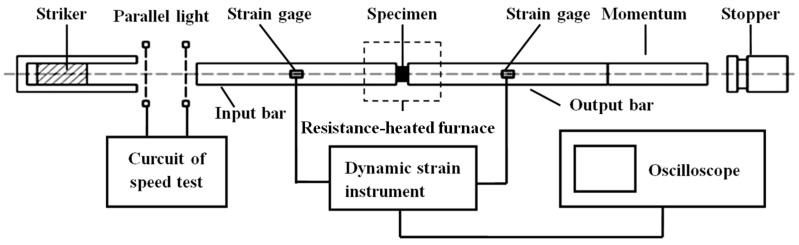
Split Hopkinson Pressure Bar (SHPB) apparatus with a resistance-heated furnace.

The set of compression samples were oriented such that the compression occurred in the normal direction (ND) of the wrought AZ31 sheet. The high strain rate compressive and elevated temperature experiments were conducted by SHPB and a resistance-heated furnace, as shown in Figure 1. More details of the procedures of the experiment are described in Zhang et al. [2,5]. In order to reveal the activation and evolution of the relevant microstructure deformation mechanism in the highly textured wrought AZ31-ND sheet, in particular, to explore the complicated competitive relationship among the microstructure deformation mechanisms under elevated temperatures at a high strain rate, compressive tests were conducted along ND, which were performed at a strain rate of 1000 s^−1^ and among the temperature range of 20–350 °C. The experimental temperature of the samples was preserved by a resistance-heated furnace with an automatic control system. The field emission scanning electronic microscope (SEM) equipped with an electron back-scattering diffraction (EBSD) detector system (Oxford HKL) was used to analyze the microstructure and micro-texture. The scanning step size, binning band, and voltage were 0.6 μm, 2 × 2, and 20 KV, respectively. The channel 5 software was used in EBSD data analysis. After being mechanically ground, the samples for EBSD mapping were electrochemically polished with 10 mL of perchloric acid and 90 mL of ethanol under 15 V at −30 °C for 120 s. In addition, in order to avoid error influence, at least 3 samples were repeated for every experimental condition. After comparing the test result of every experiment, the stress–strain curves of the wrought AZ31-TD sheets were hardly influenced by the error.

## 3. Results

### 3.1. Mechanical Responses

**Figure 2:**
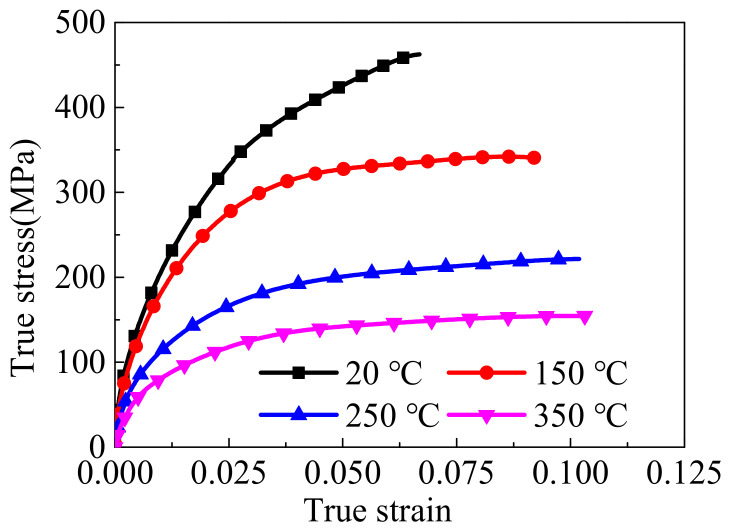
Stress–strain curves of wrought AZ31-ND in different temperatures at a strain rate of 1000 s^−1^.

The stress–strain curves of wrought AZ31-ND sheet at a strain rate of 1000 s^−1^ under different experimental temperatures are shown in Figure 2. It shows that the ultimate stresses and ultimate strain are respectively weaken and enhanced by the experimental temperature increasing, which is partly caused by the thermal softening effect [25,26]. Additionally, the thermal softening effect is caused by the reduction of *CRSS* for non-basal slip and the increased operation of non-basal glide systems with the temperature increasing [21]. Consequently, the flow stress behaviors of wrought AZ31-ND sheet are significantly affected by the experimental temperature, which has been discussed in detail in other studies [2,12]. In addition, the macro mechanical response of the material is closely reflected by the activation and evolution of the microstructure deformation mechanism. Hence, this phenomenon also indicates that the temperature has a significant effect on the microstructure deformation behavior of wrought AZ31-ND sheet.

### 3.2. Microstructure

**Figure 3:**
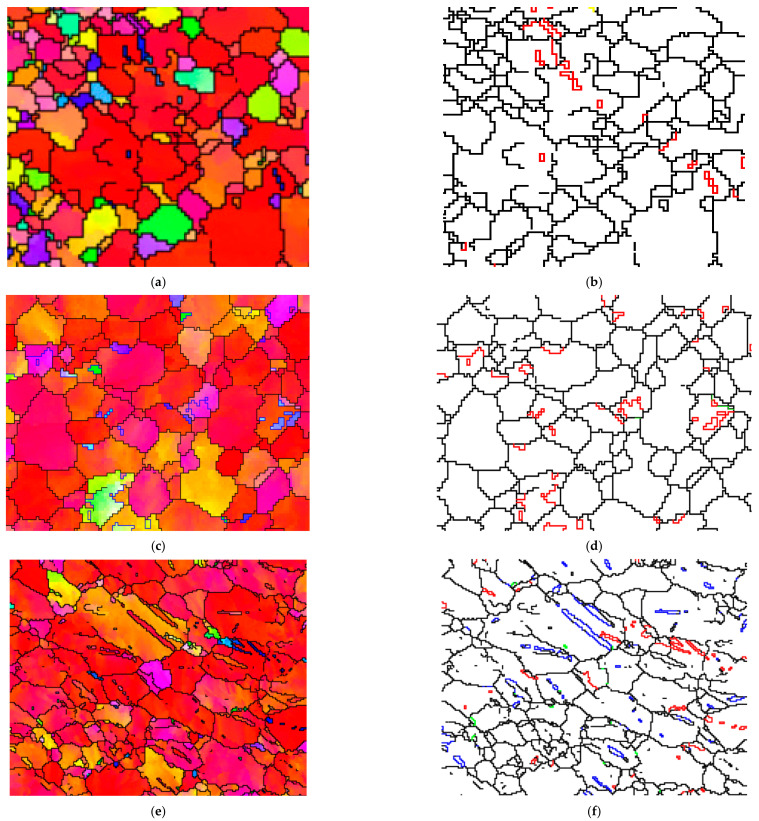

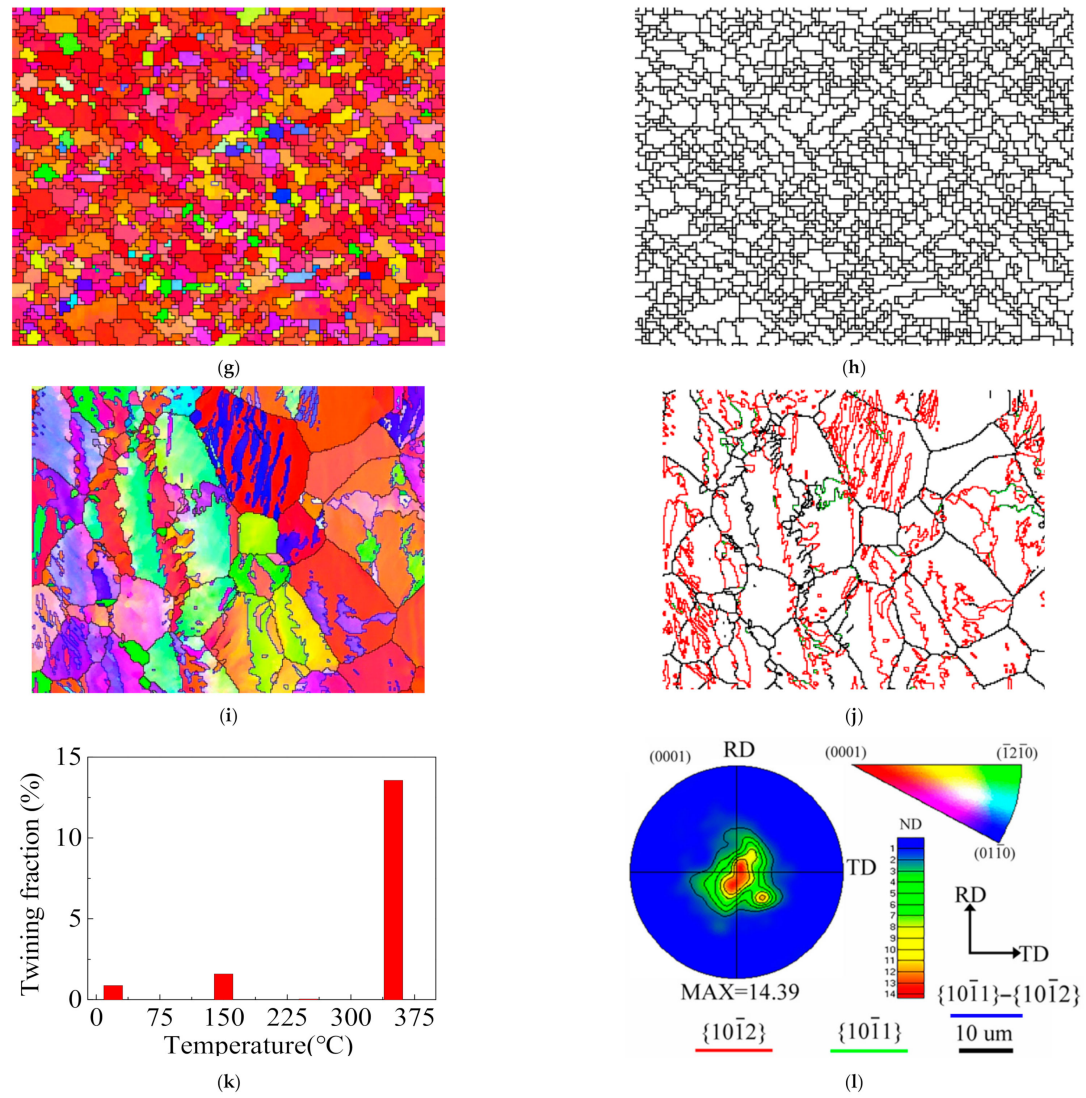
IPF maps, PF maps, BM maps, and TVF of the impacted wrought AZ31-ND sheet; the red, green, and blue line in the BM maps respectively stand for $\left\{10\bar{1}2\right\}$ tension twins, $\left\{10\bar{1}1\right\}$ contraction twins, and $\left\{10\bar{1}1\right\}$-$\left\{10\bar{1}2\right\}$ double twins: (**a**) IPF of original AZ31-ND without impact, (**b**) BM of original AZ31-ND without impact, (**c**) IPF at 20 °C, (**d**) BM at 20 °C, (**e**) IPF at 150 °C, (**f**) BM at 150 °C, (**g**) IPF at 250 °C, (**h**) BM at 250 °C, (**i**) IPF at 350 °C, (**j**) BM at 350 °C, (**k**) twinning fraction under different temperature conditions, and (**l**) PF of original AZ31-ND without impact.

**Figure 4:**
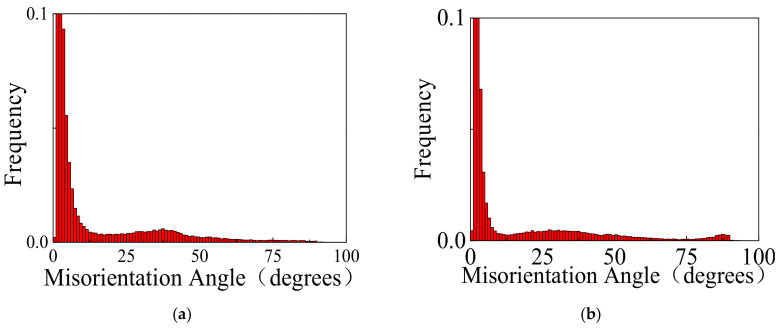

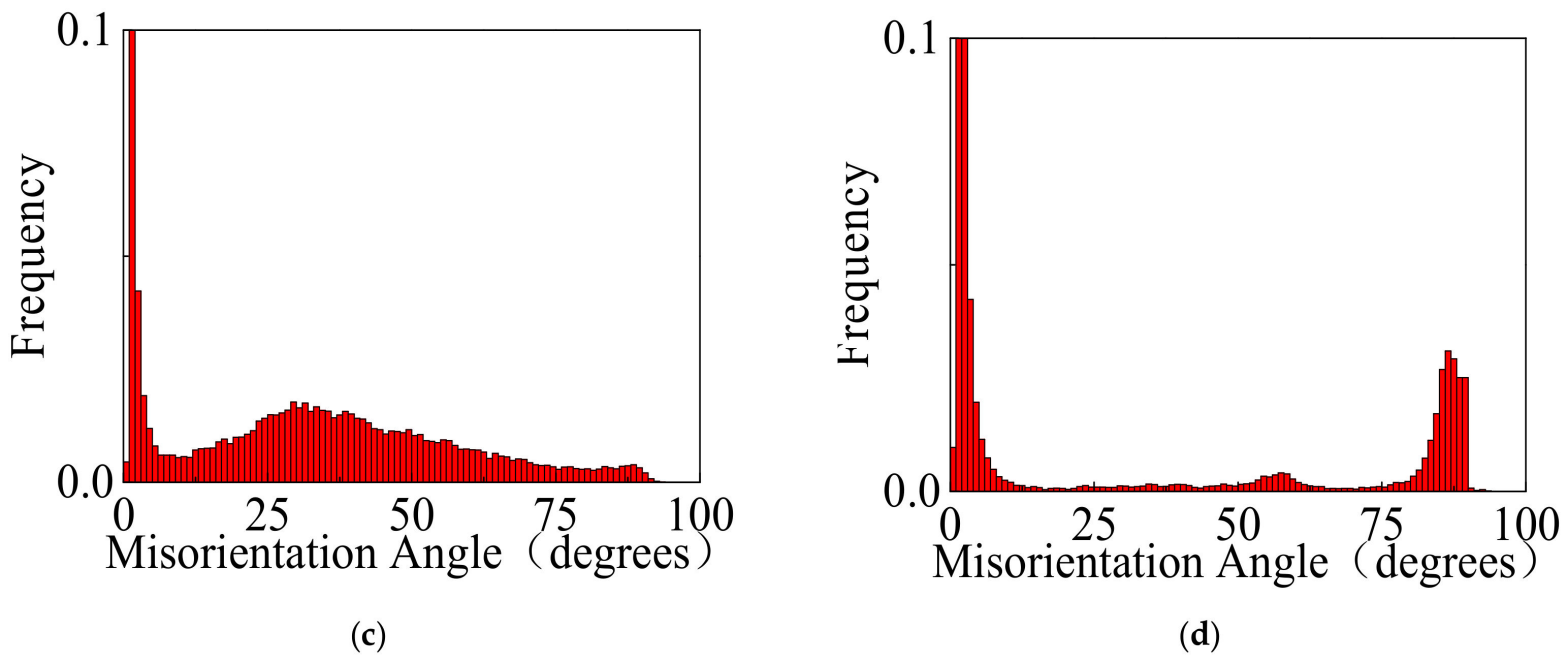
Misorientation angle distribution of impacted wrought AZ31-ND sheet: (**a**) 20, (**b**) 150, (**c**) 250, and (**d**) 350 °C.

Figure 3 presents the inverse pole figure (IPF) maps, pole figure (PF) maps, boundary misorientation (BM) maps, and twin volume fraction (TVF) of the original and impacted wrought AZ31-ND sheet at the strain rate of 1000 s^−1^ under temperatures of 20, 150, 250, and 350 °C. For the evolution tendency of the grain size under different experiment temperatures, the grain size correspondingly increased with the experimental temperature increasing. However, when the DRX mechanism was activated at 250 °C, the grain size sharply decreased. In Figure 3a,b,l, due to the c-axis of almost all grains being orientated parallel to the normal direction of wrought AZ31 sheet in the process of hot-rolling, the EBSD observation of wrought AZ31-ND sheet is a typically strong $\left\{0002\right\}$ basal plane texture. Meanwhile, the majority of the $\left\{10\bar{1}2\right\}$ tension twins are probably generated, which are gradually weakened after the heat treatment was conducted. By combining Figure 3c,d, only a small amount $\left\{10\bar{1}2\right\}$ tension twins are generated at a low temperature of 20 °C under the strain rate of 1000 s^−1^, and even no $\left\{10\bar{1}1\right\}$ contraction twins are really observed. As the compressive loading direction is parallel to the c-axis of wrought AZ31-ND sheet, the *CRSS* of the $\left\{10\bar{1}1\right\}$ contraction twins is higher, and the SF of the $\left\{10\bar{1}2\right\}$ tension twins has a relatively negative value [14,27,28]. In addition, the c-axis of most grains are still oriented to the normal direction of wrought AZ31-ND sheet. It is illustrated that the orientation of the c-axis in part grains is hardly influenced by the impact load at a strain rate of 1000 s^−1^. By comparing Figure 3a,c, it is obviously shown that the impact loading is significantly beneficial for c-axis rotation parallel to the normal direction of the wrought AZ31-ND sheet. When the experimental temperature increased to a moderately high temperature of 150 °C, the density of the $\left\{10\bar{1}1\right\}$-$\left\{10\bar{1}2\right\}$ double twins was obviously enhanced, which is shown in Figure 3e,f,k. Hence, the predominant deformation mechanism is non-uniform with $\left\{10\bar{1}1\right\}-\left\{10\bar{1}2\right\}$ double twinning. When the stress is sharply concentrated to a level for the twinning mechanism to be activated by the dislocation pile-up at the grains boundaries, particularly not only boundaries regions but also mantle regions, and as a result of low *CRSS* and high SF in $\left\{10\bar{1}1\right\}$-$\left\{10\bar{1}2\right\}$ double twins, a large number of $\left\{10\bar{1}1\right\}$-$\left\{10\bar{1}2\right\}$ double twins are generated rather than $\left\{10\bar{1}1\right\}$ contraction twins to relax the stress concentration [29,30]. In addition, the contraction twins are regarded as the c-axis of parent grains about 56°$\;\langle \bar{1}2\bar{1}0\rangle$ for the newly formed $\left\{10\bar{1}2\right\}$ tension twins. Hence, when the dynamic compression deformation is continuous, the SFs of at least two twins’ variants are positive. As a result, the $\left\{10\bar{1}2\right\}$ tension twins are preferentially nucleated inside the $\left\{10\bar{1}1\right\}$ contraction twins to form the $\left\{10\bar{1}1\right\}$-$\left\{10\bar{1}2\right\}$ double twins. The misorientation of $\left\{10\bar{1}1\right\}$-$\left\{10\bar{1}2\right\}$ double twins is 38° between the secondary $\left\{10\bar{1}2\right\}$ tension twins and the parent grains [31]. In the other case, the non-basal slip or other deformation mechanism is initially activated by the macro stress concentration in the grains, resulting in stress concentrating in the grains, which is beneficial for twinning activation by deformation to accommodate the local stress concentration [4]. According to Figure 3e, the c-axis of partly grains is reoriented to the deviation of the normal direction of the wrought AZ31-ND sheet. Moreover, when the experimental temperature is increased to 250 °C, almost all of the grains show refinement, which is caused by the dynamic recrystallization mechanism, as shown in Figure 3g,h,k. Finally, when the experimental temperature is increased to 350 °C, the refinement grains totally disappear, which are instead completed by large grains, and the majority of twins are obviously generated, as shown in Figure 3i,j. In Figure 3k, the TVF of the impacted wrought AZ31-ND sheet is higher at the experimental temperature of 350 °C rather than that of the other temperature. Figure 4 displays the misorientation angle distribution of the impacted wrought AZ31-ND sheet. In Figure 4a–c, a little misorientation of the grains is concentrated in 38° about $\langle \bar{1}2\bar{1}0\rangle$, 56° about $\langle \bar{1}2\bar{1}0\rangle$, or 86° about $\langle \bar{1}2\bar{1}0\rangle$. It is illustrated when the loading direction is parallel to the c-axis of the gains, as the twins are hardly generated at the low experimental temperature. However, for Figure 4d, when the temperature is increased to a high level, such as 350 °C, the misorientation angle of the twins is obviously concentrated at 86° about $\langle \bar{1}2\bar{1}0\rangle$ rather than that at 38° about $\langle \bar{1}2\bar{1}0\rangle$ and 56° about $\langle \bar{1}2\bar{1}0\rangle$ as a result of the low *CRSS* and high SF in the $\left\{10\bar{1}2\right\}$ tension deformation mechanism. Hence, the main type of twins is $\left\{10\bar{1}2\right\}$ tension twins. In addition, according to Figure 3i, the c-axis of the almost grains is completely reoriented to the deviation of the normal direction of the wrought AZ31-ND sheet. Hence, the direction of the c-axis in the wrought AZ31-ND sheet is significantly influenced by the higher experimental temperature. Consequently, when the compressive loading direction is parallel to the c-axis of grains in the wrought AZ31-ND sheet, the temperature increase is completely beneficial for $\left\{10\bar{1}2\right\}$ tension twinning activation, except the DRX mechanism activation.

## 4. Discussion

### 4.1. Activation of the Deformation Mechanism

**Figure 5:**
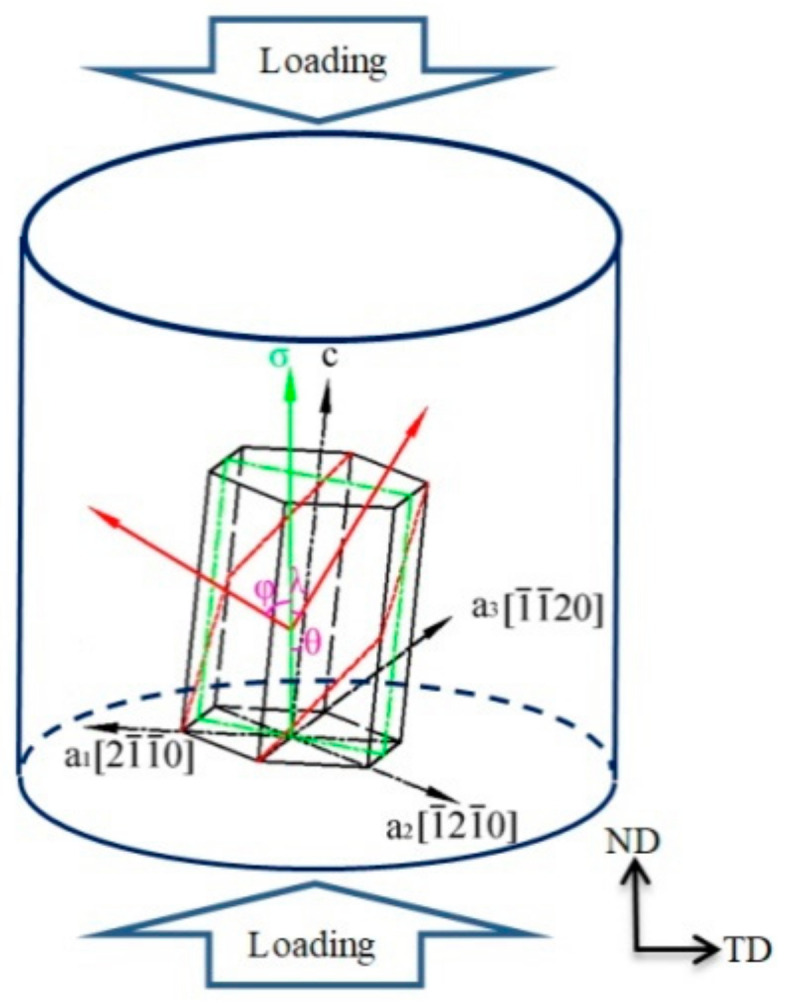
Schematic relationship among the loading direction, c-axis, a-axes, normal direction of twinning(slip) plane, and twinning(slip) direction in the AZ31-ND sample.

**Figure 6:**
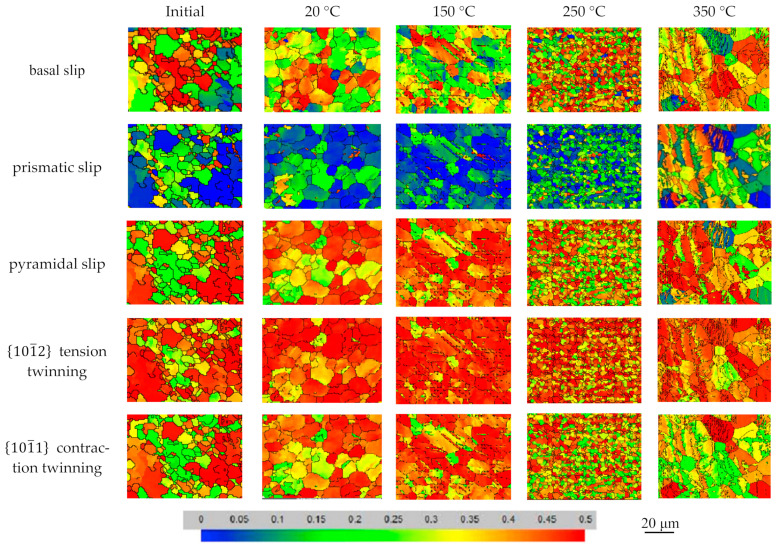
SF images of wrought AZ31-ND sheet in initial texture and different experimental temperature impacts.

**Figure 7:**
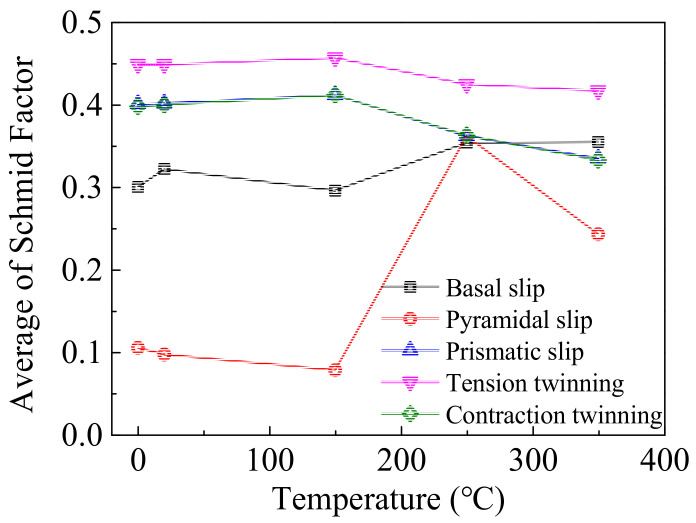
Relationship between average SF of different deformation mechanisms and experimental temperatures.

The critical activated stress of the microstructure mechanism is almost dependent on the critical resolved shear stress (*CRSS*) and the Schmid Factors (SF) [32], which is given as follows (Equation (1)):

(1) $$\sigma_{0.2}=\frac{\tau_{CRSS}}{m}$$

where *σ*_0.2_ stands for the critical equivalent stress of microstructure mechanism activation, *^τ^_CRSS_* stands for *CRSS*, and *m* stands for SF. Additionally, the SF can be established as follows (Equation (2)):

(2) m=cosφ⋅cosλ

where *φ* stands for the angle between the loading direction and the twinning (or slip) plane normal and *λ* stands for the angle between the loading direction and the twinning (or slip) direction, which are shown in Figure 5. For the indices direction of the two-four-dimensional Miller–Bravais system, such as $\left\{h,k,i,l\right\}-\langle u,v,t,w\rangle$, the twinning or slip plane normal is calculated as follows (Equation (3)):

(3) $$\left[u,v,t,w\right]=\left[h,k,i,\frac{3l}{2}{\left(\frac{c}{a}\right)}^{2}\right]$$

where a and c are the lattice constants, and, generally, the c/a axial ratio is 1.624 [31]. Finally, the cos*φ* and cos*λ* can be directly calculated using Equation (4):

(4) $$\cos\varphi (\lambda )=\frac{V_{1}\cdot V_{2}}{\left|V_{1}\right|\cdot \left|V_{2}\right|}=\frac{u_{1}u_{2}+V_{1}V_{2}+\frac{1}{2}(u_{1}V_{2}+u_{2}V_{1})+\frac{1}{3}w_{1}w_{2}{\left(\frac{c}{a}\right)}^{2}}{\sqrt{u_{1}{}^{2}+V_{1}{}^{2}+u_{1}V_{1}+\frac{V_{1}{}^{2}}{3}{\left(\frac{c}{a}\right)}^{2}}\times \sqrt{u_{2}{}^{2}+V_{2}{}^{2}+u_{2}V_{2}+\frac{V_{2}{}^{2}}{3}{\left(\frac{c}{a}\right)}^{2}}}$$

where *V*_1_ $\left[u_{1},v_{1},t_{1},w_{1}\right]$ is the twinning (slip) plane normal or twinning (slip) direction and *V*_2_ $\left[u_{2},v_{2},t_{2},w_{2}\right]$ is the loading direction. The average SF in different deformation mechanisms of wrought AZ31-ND sheet is listed in Table 2, which is the corresponding numerical expression in Figure 6 using Channel 5 software. As the direction of loading is almost opposite to the tensile direction of the c-axis, the SF value of the $\left\{10\bar{1}2\right\}$ tension twinning mechanism is negative, which is marked with a symbol of “*”. In order to analyze the high-temperature impacting effect on the average SF of different deformation mechanisms, the relationship between the average SF and experimental temperature is shown in Figure 7. As the average SF of $\left\{10\bar{1}2\right\}$ tension twinning is a negative value, it is useless to analyze the average SF effect on the $\left\{10\bar{1}2\right\}$ tension twinning mechanism. By comparing the SF of different deformation mechanisms in the initial and 20 °C impact post, when the loading direction is parallel to the normal direction of wrought AZ31-ND sheet, the average SF of basal slip, pyramidal slip, and $\;\left\{10\bar{1}1\right\}$ contraction twinning is respectively increased from 0.3 to 0.322, 0.401 to 0.402, and 0.398 to 0.4. Additionally, the average SF of prismatic slip is decreased from 0.105 to 0.097. Hence, according to the SF analysis, the loading is positively affected after activation of basal slip, pyramidal slip, and $\;\left\{10\bar{1}1\right\}$ contraction twinning but negatively affected after activation of prismatic slip. When the experimental temperature is increased to 150 °C, the average SF of basal slip and prismatic slip is respectively decreased from 0.322 to 0.296 and 0.097 to 0.078. Additionally, the average SF of pyramidal slip and $\left\{10\bar{1}1\right\}$ contraction twinning is respectively increased from 0.402 to 0.412 and 0.4 to 0.412. Hence, according to the SF analysis, the experimental temperature is negatively affected after activation of basal slip and prismatic slip but positively affected after activation of pyramidal slip and $\left\{10\bar{1}1\right\}$ contraction twinning. Furthermore, the experimental temperature is 250 °C and the DRX is obviously present. The orientation of the c-axis in the refinement grains is a randomly redistribution as a result of the impact loading and high temperature. Hence, the average SF of basal slip, prismatic slip, pyramidal slip, and $\;\left\{10\bar{1}1\right\}$ contraction twinning is almost equivalent to each other. Until the experimental temperature is 350 °C, the phenomenon of grain growth is gradually present. The average SF of basal slip and prismatic slip is respectively increased from 0.296 to 0.355 and 0.078 to 0.243. Additionally, the average SF of pyramidal slip and $\left\{10\bar{1}1\right\}$ contraction twinning is respectively decreased from 0.412 to 0.335 and 0.412 to 0.333. Hence, according to the SF analysis, the grain growth is positively affected by the activation of basal slip and prismatic slip but negatively affected by the activation of pyramidal slip and $\left\{10\bar{1}1\right\}$ contraction twinning.

**Figure 8:**
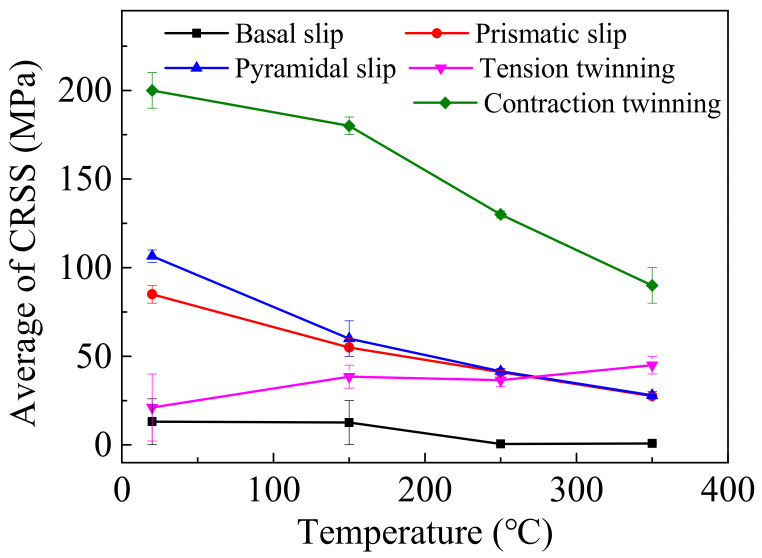
Relationship between *CRSS* of different deformation mechanism and experimental temperature.

**Figure 9:**
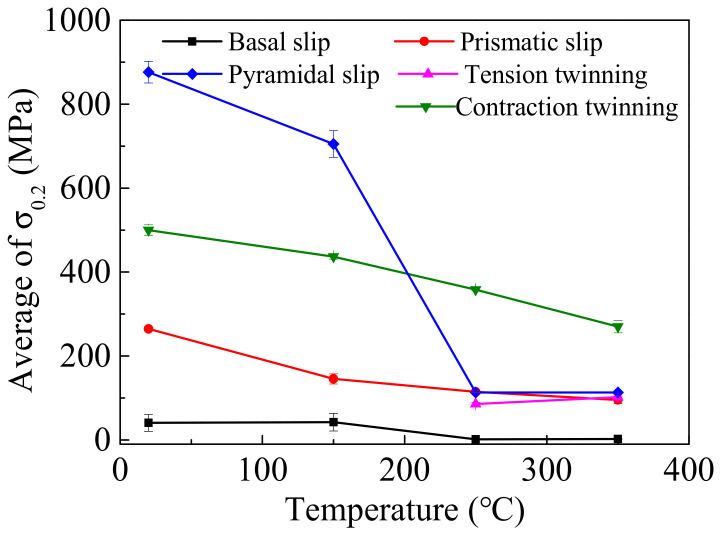
Relationship between *σ*_0.2_ of different deformation mechanisms and experimental temperatures.

Particularly, the activation of the deformation mechanism is predominantly determined by *CRSS* and SF. The reported *CRSS* of different deformation mechanisms in different experimental temperatures is listed in Table 3 [7,14,33,34,35,36], which corresponds to the curves in Figure 8. According to Equation (1), the *σ*_0.2_ of different deformation mechanism can be calculated and listed in Table 4, which corresponds to the curves in Figure 9. It is worth mentioning that when the experimental temperature is higher than 250 °C, the c-axis of most grains is randomly reoriented by DRX mechanism activation or grain growth. Thus, the average SF of $\left\{10\bar{1}2\right\}$ tension twinning has a positive value. Consequently, the *σ*_0.2_ of $\left\{10\bar{1}2\right\}$ tension twinning is also necessary for the calculation. In Figure 9, as the *σ*_0.2_ of different deformation mechanisms decreased with the experimental temperature increasing, the deformation mechanism activation of wrought AZ31-ND sheet is positively driven by the temperature. When the experimental temperature is lower than 250 °C, the *σ*_0.2_ of basal slip and pyramidal slip is relatively lower than the others, which can all be activated. However, the basal slip can only provide two independent slip systems, which is relatively limited for large plastic deformation of the wrought AZ31-ND sheet [37]. Therefore, the pyramidal <a> and <c+a> slip are particularly the predominant deformation mechanisms coordinating the inhomogeneous plastic deformation and relaxing the shear strain concentration around the double twins through the deformation component in the <c> direction, which can provide five independent slip systems [38,39]. When the temperature is higher than 350 °C, the c-axis of grains is randomly reoriented. The *σ*_0.2_ of $\left\{10\bar{1}2\right\}$ tension twinning is larger than that of basal slip but close to that of pyramidal <a> and <c+a> slip. In addition, the majority of the inhomogeneous plastic strain is continuous during the high-temperature dynamic compressive behavior of the wrought AZ31-ND sheet, which is necessary for coordinate deformation by the pyramidal <a> and <c+a> slip. However, the *σ*_0.2_ of $\left\{10\bar{1}2\right\}$ tension twinning at 250 °C is lower than that of pyramidal <a> and <c+a> slip, which is beneficial for a certain competitive advantage of $\left\{10\bar{1}2\right\}$ tension twinning activation [12,40]. Hence, large $\left\{10\bar{1}2\right\}$ tension twins are generated in the grains, which corresponds to the result of Figure 3j.

### 4.2. Dynamic Recrystallization

**Figure 10:**
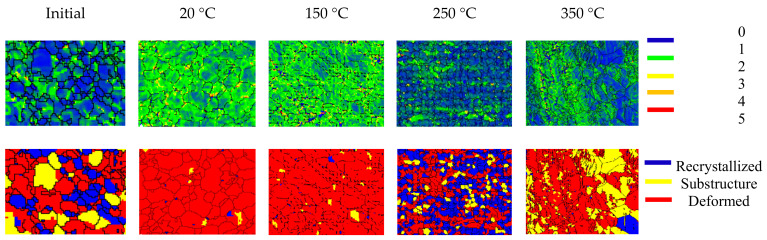
Kernel average misorientation map and distribution maps of the recrystallized, substructure, and deformed grains in different conditions.

In order to analyze the DRX mechanism of the wrought AZ31-ND sheet at a strain rate of 1000 s^−1^ in the experimental temperature range from 20 to 350 °C, the kernel misorientation maps and the distribution maps of recrystallized, substructure, and deformed grains are shown in Figure 10. For the initial status, the strain concentration regions are randomly distributed in the grains, which is enhanced at the deformed grain boundaries. During the rolled process and heat treatment of the wrought AZ31-ND sheet, several substructure or DRX grains gradually nucleate. Therefore, the kernel misorientation concentration regions are weakened. After the high temperature (20–150 °C) impact, the majority of the substructure and DRX grains are completely converted to deformed grains. Hence, the kernel misorientation concentration regions are obviously enhanced, especially in the grain boundaries. Until the experimental temperature is 250 °C, the DRX mechanism of the wrought AZ31-ND is completely activated. According to Figure 3f, $\left\{10\bar{1}1\right\}$ contraction twins and $\left\{10\bar{1}1\right\}$-$\left\{10\bar{1}2\right\}$ double twins provide a special angular relationship with a basal plane, such as 56° for $\left\{10\bar{1}1\right\}$ contraction twins and 38° for $\left\{10\bar{1}1\right\}$-$\left\{10\bar{1}2\right\}$ double twins, which are regarded as effective nucleation sites for inducing the TDRX mechanism activation [41]. In addition, as the higher strain energy is almost inhomogeneously stored in $\;\left\{10\bar{1}1\right\}$-$\left\{10\bar{1}2\right\}$ double twins, the formation of TDRX is induced more easily by $\left\{10\bar{1}1\right\}$-$\left\{10\bar{1}2\right\}$ double twins than that by the primary $\left\{10\bar{1}2\right\}$ tension twins [42]. However, the volume fraction of twins is only 1.5%, as shown in Figure 3k. It is implied that there are not enough nucleation sites of TDRX. According to Figure 9, before activation of the DRX mechanism of wrought AZ31-ND sheet, the pyramidal <a> and <c+a> slip are particularly the predominant deformation mechanism. Hence, the DRX mechanism type of the wrought AZ31-ND sheet is dedicated to RDRX [18,43]. Meanwhile, the kernel misorientation concentration regions are weakened again as a result of the substructure, large number of RDRX grains nucleating, and limited number of TDRX grains nucleating. When the experimental temperature is increased to 350 °C, the majority of $\left\{10\bar{1}2\right\}$ tension twins are generated in the bigger deformed grains. The kernel misorientation concentration regions are enhanced again, especially in $\left\{10\bar{1}2\right\}$ tension twin boundaries. Consequently, the kernel misorientation concentration regions are obviously enhanced by deformed grains and $\left\{10\bar{1}2\right\}$ tension twins but weakened by substructure grains and DRX grains.

## 5. Conclusions

(1) During the high-temperature dynamic deformation, the c-axis of grains is gradually reoriented parallel to the normal direction of the wrought AZ31-ND sheet with the temperature increasing, except the DRX mechanism is activated or grains grow up, which is completely beneficial for $\left\{10\bar{1}2\right\}$ tension twinning activation.
(2) At lower temperatures (≤250 °C), when the loading direction is parallel to the c-axis of the grains, the pyramidal <a> and <c+a> slip are the particularly predominant deformation mechanism. At higher temperatures (≥250 °C), $\left\{10\bar{1}2\right\}$ tension twinning is the predominant deformation mechanism. Otherwise, the predominant type of the DRX mechanism of the wrought AZ31-ND sheet is RDRX.
(3) The kernel misorientation concentration regions are always enhanced by deformed grains, especially by $\left\{10\bar{1}2\right\}$ tension twins, but weakened by substructure grains and DRX grains.

## Figures and Tables

**Table 1:** Chemical composition of AZ31 magnesium alloy.

Composition	Al	Mn	Zn	Fe	Si	Be	Cu	Mg
Content	3.19	0.334	0.81	0.005	0.02	0.01	0.005	Bal.

**Table 2:** Average of SF in different deformation mechanisms.

Deformation Mechanisms	Initial	20 °C	150 °C	250 °C	350 °C
basal slip	0.3	0.322	0.296	0.354	0.355
prismatic slip	0.105	0.097	0.078	0.362	0.243
pyramidal slip	0.401	0.402	0.412	0.362	0.335
$\left\{10\bar{1}2\right\}$ tension twinning	0.449 *	0.449 *	0.457 *	0.425	0.417
$\left\{10\bar{1}1\right\}$ contraction twinning	0.398	0.4	0.412	0.363	0.333

^“^*^”^ stands for negative value.

**Table 3:** *CRSS* (MPa) reported of different deformation mechanisms [7,14,33,34,35,36].

Deformation Mechanisms	20 °C	150 °C	250 °C	350 °C
basal slip	0.2–26	0.2–25	0.2–0.81	0.2–0.8
prismatic slip	80–90	50–65	40–42	26–29
pyramidal slip	103–110	50–70	40–43	26–38
$\left\{10\bar{1}2\right\}$ tension twinning	2.2–40	32–45	33–40	40–45
$\left\{10\bar{1}1\right\}$ contraction twinning	190–210	175–185	128–132	80–100

**Table 4:** *σ*_0.2_ calculation of different deformation mechanisms.

Deformation Mechanisms	20 °C	150 °C	250 °C	350 °C
basal slip	21–61	22–64	1–2	2
prismatic slip	851–902	673–737	112–115	110–116
pyramidal slip	261–269	133–158	113–117	87–104
$\left\{10\bar{1}2\right\}$ tension twinning	-	-	82–90	99–105
$\left\{10\bar{1}1\right\}$ contraction twinning	488–513	431–443	355–361	255–285

## Data Availability

The data presented in this study are available upon request from the corresponding author.
